# Endothelial progenitor cells in pregnancy-related diseases

**DOI:** 10.1042/CS20230853

**Published:** 2023-11-21

**Authors:** Yangyang Chen, Gui Wan, Zeyun Li, Xiaoxia Liu, Yin Zhao, Li Zou, Weifang Liu

**Affiliations:** 1 Department of Obstetrics and Gynecology, Union Hospital, Tongji Medical College, Huazhong University of Science and Technology, Wuhan 430022, China; 2 Department of Neurosurgery, Peking Union Medical College Hospital, Chinese Academy of Medical Sciences and Peking Union Medical College, Beijing 100730, China; 3 The First Clinical School of Tongji Medical College, Huazhong University of Science and Technology, Wuhan 430022, China

**Keywords:** Endothelial progenitor cell, Neovascularization, Preeclampsia, Pregnancy-related complication

## Abstract

Placental neovascularization plays a crucial role in fetomaternal circulation throughout pregnancy and is dysregulated in several pregnancy-related diseases, including preeclampsia, gestational diabetes mellitus, and fetal growth restriction. Endothelial progenitor cells (EPCs) are a heterogeneous population of cells that differentiate into mature endothelial cells, which influence vascular homeostasis, neovascularization, and endothelial repair. Since their discovery in 1997 by Asahara et al., the role of EPCs in vascular biology has garnered a lot of interest. However, although pregnancy-related conditions are associated with changes in the number and function of EPCs, the reported findings are conflicting. This review discusses the discovery, isolation, and classification of EPCs and highlights discrepancies between current studies. Overviews of how various diseases affect the numbers and functions of EPCs, the role of EPCs as biomarkers of pregnancy disorders, and the potential therapeutic applications involving EPCs are also provided.

## Introduction

Placental neovascularization plays a crucial role in fetomaternal circulation and ensures optimal exchange of nutrients, gases, and metabolic waste throughout pregnancy [1]. The failure of these processes has been associated with high perinatal morbidity and mortality and key findings indicate that many pregnancy-associated disorders are associated with dysregulated placental neovascularization. Vasculogenesis and angiogenesis, the main mechanisms of neovascularization, drive the formation of the placenta’s vascular network [2]. During vasculogenesis, the first primitive vascular plexus is formed via the differentiation of primitive progenitors, which is thought to take place only during embryogenesis, whereas the subsequent step, angiogenesis, involves the formation of new vessels from pre-existing ones and is thought to occur during embryonic development and in postnatal life. These processes are crucial for the establishment and development of placental blood vessels [3], and their failure are key causes of placental disorders, including preeclampsia (PE), intrauterine growth restriction (IUGR), and gestational diabetes mellitus (GDM) [3–5].

Endothelial progenitor cells (EPCs) are key factors in vasculogenesis and angiogenesis with the ability to mobilize, migrate, and integrate into new vessels, where they can differentiate into mature endothelial cells (ECs) [6–8]. EPCs are involved in various vascular disorders, such as myocardial infarction, stroke, limb ischemia, impaired wound healing, atherosclerosis, diabetic microvasculopathy, ischemic retinopathy, and pulmonary arterial hypertension [9–12]. Several therapeutic strategies, including EPC transplantation, gene reprogramming, and drugs, have been proposed for the treatment of pregnancy-related diseases. However, few in-depth studies have investigated the role of EPCs in pregnancy-related disorders, probably because of the intricacy of the relationship between EPCs and neovascularization in many pregnancy complications. Here, we critically discuss the mechanisms by which EPCs influence pregnancy-related diseases, as well as their potential diagnostic and therapeutic applications.

## Discovery, isolation, origin, and classification of EPCs

EPCs were first described in 1997 by Asahara et al. as circulating adult EPCs that shared surface antigens with ECs and contributed to angiogenesis in ischemic tissues [13]. EPCs were originally described as CD34+ (human), Fetal liver kinase 1 (Flk-1, also known as vascular endothelial growth factor receptor 2 [VEGFR2])+, or kinase insert domain receptor (KDR) (murine) mononuclear blood cells. Additionally, Ficoll density gradient centrifugation (DGC) was proposed for the isolation of EPCs from human peripheral blood (PB). The definition, isolation, and classification of EPCs have been updated severally, and there has been intense controversy about the origin of EPCs.

**Figure 1:**
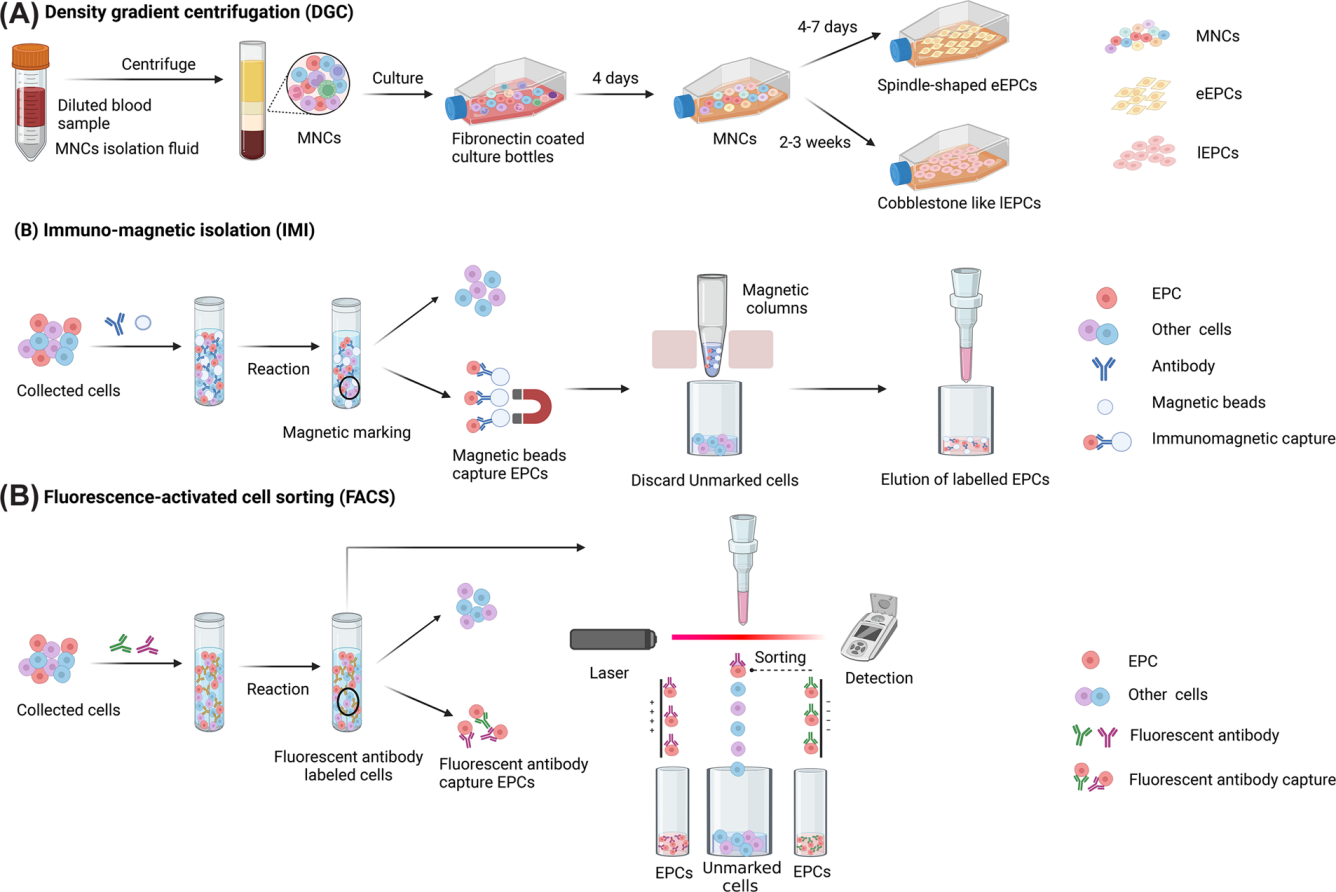
Different approaches to isolate EPC (**A**) Density gradient centrifugation (DGC). (**B**) Immuno-magnetic isolation (IMI). (**C**) Fluorescence-activated cell sorting (FACS).

Different approaches have been used to isolate EPCs (Figure 1), including DGC, immunomagnetic isolation (IMI), and fluorescence-activated cell sorting (FACS) [13–15]. In DGC, mononuclear cells (MNCs) are isolated and cultured on fibronectin-coated dishes in EBM-2 media supplemented with the EGM-2 bullet kit for 4 days. Nonadherent cells are washed out using PBS, leaving MNCs on the culture dish. When cultured for 4–7 days, spindle-shaped early EPCs (eEPCs) are obtained [16], whereas culturing for 2–3 weeks produces cobblestone-like, late EPCs (lEPCs) [17]. Other studies have isolated EPCs by culturing adherent MNCs for 24 or 48 h and then replating the nonadherent cells [18]. In this way, the first adherent cells containing monocytes, macrophages, and circulating mature ECs are removed to obtain adherent EPCs, which are collected later. IMI isolates EPCs based on cell surface markers [19]. Commonly used cell surface markers include CD133, CD34, CD90, CD31, and CD14. EPC isolation using FACS relies on staining cells with fluorescent antibodies against one or more cell surface markers and then sorting target cells based on their fluorescence intensity [20]. For FACS, the most frequently used markers are CD34, VEGFR2, CD45, CD31, CD144, and CD146. A hybrid assay that first isolates the cells using IMI or DGC followed by multiparameter FACS analysis has also been developed [21]. These methods have unique advantages and disadvantages and there is debate about which one is best for EPC isolation. Indeed, while DGC is considered a crude method that does not morphologically determine whether the isolated cells are EPCs, IMI, and FACS, which rely on cell–surface antigens, can isolate EPCs more accurately. Additionally, IMI can recover very low cell numbers that may be below the threshold of detection through FACS. Moreover, IMI, but not FACS, allows the morphological analysis of EPCs. However, IMI can only capture EPCs based on one antibody, whereas FACS can use multiple markers to isolate EPCs and evaluate multiple EPC phenotypes simultaneously. Although in hybrid assays the individual techniques can complement each other, there is no consensus about EPC surface markers, which has limited the clinical application of these technologies.

EPCs can be isolated from the bone marrow (BM), PB, and umbilical cord blood (UCB) [22]. They are also located in vessel walls, umbilical cord, adipose tissue, cardiac tissue, placenta, spleen, and neural tissue [23]. It has been reported that in response to ischemic injury, circulating EPCs (cEPCs) originate from the BM mobilize and home in injured vascular sites. However, a recent finding indicates that cEPCs are derived from vessel walls or the endothelium, and not from the BM [8,24]. Moreover, some studies have suggested that circulating ECs (CECs) do not correspond to actual cEPCs but include mature ECs and monocyte/macrophage-derived cells [25]. When vessels are damaged, most detached CECs are exposed to inflammatory cytokines and might undergo apoptosis or necrosis, and few viable CECs exhibit cEPC-like properties [26]. EPCs are also reported to localize in adipose tissue [27], but it is unclear if these are the true origin of cEPCs. However, the main view is that cEPCs originate from the BM. UCBs are reported to be the largest source of stem cells and currently, there are >450,000 unrelated UCB banks [28]. UCBs contain more EPCs than PB and EPCs isolated from UCBs have key advantages over those from other sources. For example, UCB-derived EPCs exhibit long telomeres and abundantly express genes involved in the cell cycle and blood vessel development, which are associated with high levels of telomerase activity and high proliferation potential [29]. Additionally, PB-derived EPCs are reported to form unstable blood vessels, which might regress within 3 weeks, whereas UCB-derived EPCs form stable blood vessels that can last for more >4 months [30]. Moreover, UCB-derived EPCs can be obtained noninvasively, without risk to the donor, and their transplantation is associated with a lower risk of infection and graft versus host disease.

There are several EPC subtypes, including eEPCs and lEPCs. eEPCs and lEPCs have different phenotypes, which are defined according to their morphological appearance in culture (Table 1). eEPCs are also called circulation angiogenetic cells or colony forming unit-endothelial cells (CFU-ECs or CFU-Hill), while lEPCs are also known as outgrowth endothelial cells (OECs) or endothelial colony-forming cells (ECFCs) [31]. EPCs can also be classified as ‘hematopoietic’ or ‘nonhematopoietic EPCs’, which share a common precursor. EPC surface markers, including proteins and carbohydrates attached to the cell membrane, and play important roles in cell identification. EPCs were first described by Asahara et al. as cells that co-express the hematopoietic stem cell marker CD34 and the EC marker VEGFR2 in 1997 [13]. However, because mature ECs also co-express CD34 and VEGFR2, more specific EPC markers were needed. Another hematopoietic stem cell marker, CD133, and the monocyte marker, CD14, which were reported to be expressed on eEPCs but not on mature ECs, were proposed as suitable markers for immature EPCs [32]. Therefore, eEPCs can be identified by the three kinds of cellular surface markers, namely hematopoietic (CD133+, CD45+), monocyte (CD14+) and endothelial ones (CD34+, VEGFR2+, CD31+). On the other hand, EPCs with negative expression of hematopoietic antigens (CD45, CD177, and CD133) and monocyte marker (CD14) and positive expression of endothelial markers (CD31, VEGFR2, CD34, VE-cadherin, E-selectin, and vWF) were defined as lEPCs [31,33–35]. Both eEPCs and lEPCs can take up acetylated low-density lipoprotein (ac-LDL) and bind Ulex europaeus agglutinin I (UEA-1) [36]. Although eEPCs do not incorporate into the vasculature, they abundantly secrete paracrine factors, such as angiogenic cytokines and chemokines, which promote the adherence and accumulation of cells in the surroundings of impaired vessels, thereby indirectly accelerating neovascularization [37]. Notably, eEPCs may also participate in inflammatory processes by differentiating into macrophages [38,39]. However, lEPCs easily form vessels and display strong spontaneous angiogenic potential, with a high proliferation and migration capacity. When blood vessels are injured *in vivo*, lEPCs rapidly migrate to the systemic vasculature, where they home, integrate into the damaged vessels, differentiate into ECs, and form a stable vascular structure, thereby directly repairing injured vessels [16]. Moreover, using lEPCs for EPC-based treatment of microvascular damage has several advantages over eEPCs [40]. Thus, EPCs are not a single cell type and vary greatly in different types.

**Table 1:** Characteristics of eEPC and lEPC

Characteristics	eEPC	lEPC
Other names	CACs, CFU-ECs, CFU-Hill	OECs, ECFCs
Shape	Spindle	Cobblestone
Colony	4–7 days	2–3 weeks
Peak growth pattern	Between 2nd and 3rd week	Between 4th and 8th week
Life time	4 weeks	12 weeks
Hematopoietic antigen	Positive (CD133+, CD45+)	Negative (CD45-, CD177-, CD133-)
Markers		
Monocyte marker	Positive (CD14+)	Negative (CD14-)
Endothelial marker	Positive (CD34+, VEGFR2+, CD31+)	Positive (CD31+, VEGFR2+, CD34+)
Others		Positive (VE-cadherin+, E-selectin+, vWF+)
Specific properties	Bind UEA-I lectin and take up LDL	Bind UEA-I lectin and take up LDL
Functions		
Neovascularization	Indirectly, paracrine factors	Directly, angiogenic potential
Others	Immunity, inflammation	None

eEPC, Early endothelial progenitor cell; lEPC: late endothelial progenitor cell.

## The roles and mechanisms of EPCs during placenta formation

**Figure 2:**
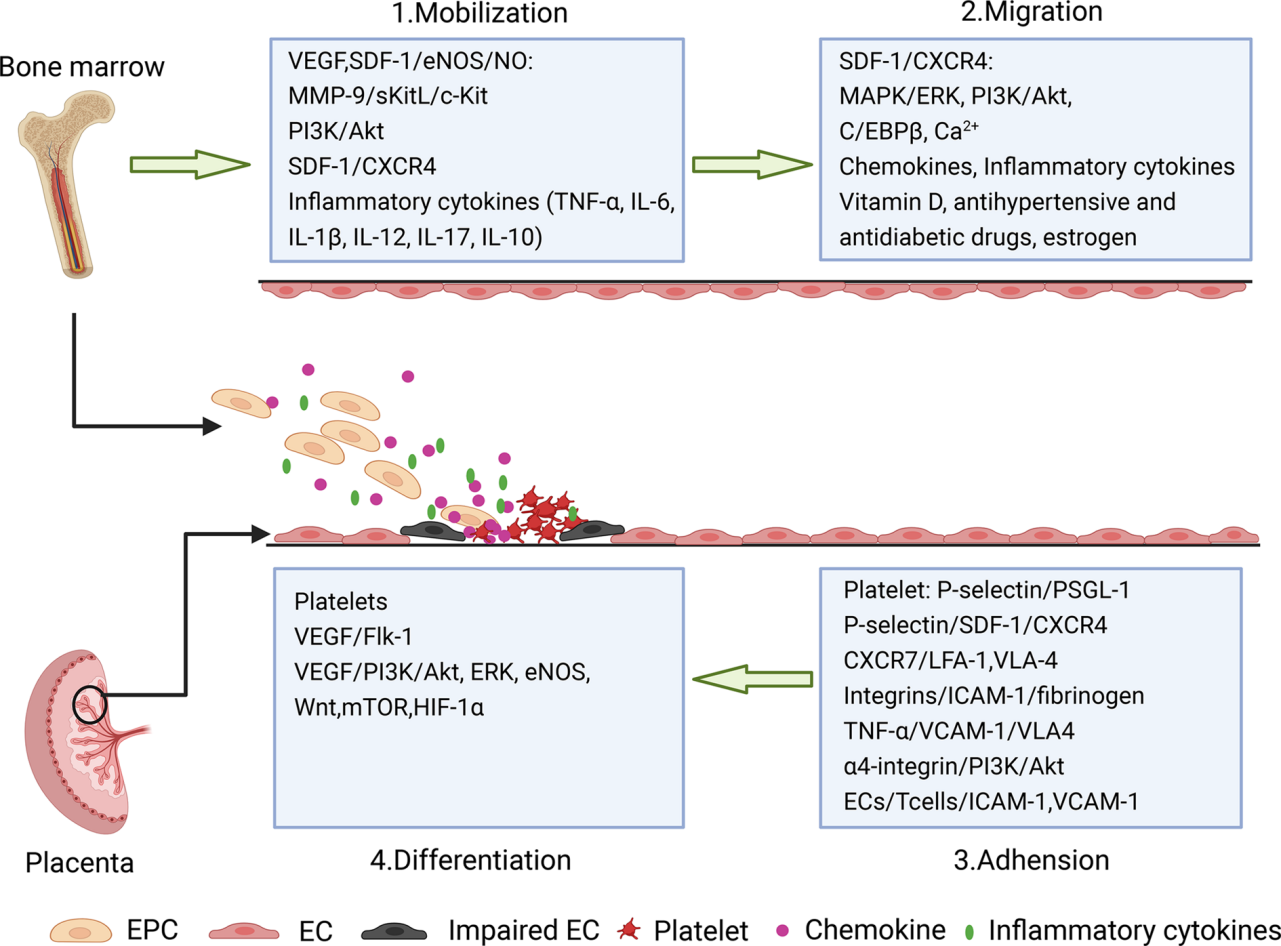
The multiple steps of EPCs’ biology during angiogenesis

In humans, angiogenesis and revascularization, which begin on gestation day 18–20 and continue throughout pregnancy, are essential for the optimal supply of nutrients and oxygen to the fetus [41]. EPCs can migrate and adhere to the neovascularization site, proliferate rapidly, and differentiate into mature ECs. Studies in animals and humans have demonstrated the involvement of EPCs in angiogenesis during placenta formation [42]. Generally, EPCs promote neovascularization through mobilization from the BM, migration to the site of neovascularization, and differentiation into mature ECs (Figure 2).

### Functions

#### Mobilization

EPC mobilization from the BM to the peripheral circulation is a crucial step in postnatal neovascularization. However, the mechanism underlying EPC mobilization is not fully understood. EPCs can mobilize in response to various growth factors and chemokines, which bind to specific receptors to activate various pathways, including VEGF/VEGFR2, stromal cell-derived factor-1 (SDF-1)/CXC-chemokine receptor 4 (CXCR4), and basic fibroblast growth factor (FGF)/FGFR signaling pathways.

VEGFs, the most important family of proangiogenic agents include six family members, VEGF, placenta growth factor (PlGF), VEGF-B, VEGF-C, VEGF-D, and VEGF-E [43,44]. VEGF is a key driver of neovascularization and can bind to the VEGF receptors, VEGFR1 (Flt-1), VEGFR2, and VEGFR-3 (Flt-4), which are expressed on vascular ECs and the lymphatic endothelium [45]. VEGFR1 and VEGFR2 are involved in VEGF-mediated EPC mobilization and recruitment [46,47]. VEGF and one of its downstream signaling factors, endothelial nitric oxide synthase (eNOS), have been shown to stimulate EPC mobilization from the BM to the PB [48]. In addition, nitric oxide (NO) is known to regulate various physiological functions, including angiogenesis, thrombosis, vasoconstriction, and vasodilatation. NO influences EPC mobilization and is rapidly produced by eNOS, an effector molecule of the VEGF/VEGFR2/PI3K/Akt signaling axis [49,50]. The elevated NO levels activate matrix metalloproteinase-9 (MMP-9) and convert membrane-bound kit ligand (mkitL) into soluble kit ligand (sKitL). These ligands can bind to the c-kit receptor on EPCs, with skitL binding suppressing mkitL binding and triggering EPC mobilization. Along with increased local protease activity and circulating SDF-1 levels, stromal cell–EPC interactions are weakened and EPCs are mobilized out of the BM along the SDF-1 concentration gradient. The levels of SDF-1 (CXCL12), a potent chemokine, increase with rising risk factors, such as inflammation, hypoxia, and ROS [51,52]. The SDF-1 receptor, CXCR4, is highly expressed by hematopoietic progenitors and EPCs [53]. Furthermore, the interaction between SDF-1 and CXCR4 not only initiates EPC mobilization from BM but also promotes EPC recruitment and adhesion to the neovascularization site [54]. Additionally, several inflammatory factors, including interleukin (IL)-6, IL-1β, IL-12, IL-10, and tumor necrosis factor-α (TNF-α), modulate EPC mobilization, migration, and adhesion. Matrix metalloproteinases (MMPs) also act as inflammatory cytokines during vascular formation or remodeling, and they can be activated by TNF-α and ILs. In turn, MMPs degrade the extracellular matrix (ECM), thereby facilitating the migration and recruitment of inflammatory cells and EPCs, as well as the cleavage of cell surface receptors and other non-ECM molecules, mediating adhesion of cells in the vessel wall, which are involved in inflammatory processes [55]. Taking TNF-α, IL-17, and IL-18 as examples, TNF-α activates MMP-9 expression at the transcriptional level, while IL-17 and IL-18 also stimulate MMP-9 expression via the NF-κB and the activator protein 1 signaling pathways. Upon mobilization from the BM niche, EPCs invade the ECM and engraft at the target site, where they proliferate and differentiate. However, increasing evidence indicates that a transient restricted inflammatory response may stimulate EPC mobilization, while persistent or excessive inflammatory stimuli may have deleterious effects, resulting in decreased EPC mobilization into the circulation [56]. High TNF-α levels suppress the number and function of circulating EPCs. Although the mechanisms regulating this effect are still unclear, prolonged inflammation in the BM may exhaust the EPC pool.

#### Migration

SDF-1, a member of the chemokine superfamily, regulates multiple physiological and pathological processes, including angiogenesis, immunity, and inflammation [51,57]. SDF-1/CXCR4 signaling activates downstream pathways, such as MAPK/ERK and PI3K/AKT, resulting in calcium mobilization and cytoskeletal reorganization [53,58]. The abundant release of chemokines and inflammatory cytokines, such as CINC-2β, IL-6, IL-10, IL-1β, and TNF-α at injured and inflamed sites mainly activates PI3K/AKT and MAPK/ERK signal pathways, which drives EPC migration to these sites [40,53,59]. SDF-1 is also reported to affect calcium flux and cytoskeletal reorganization in EPCs, thereby regulating their migration. Recent studies indicate that vitamin D [60], antihypertensive drugs [61], antidiabetic drugs [62], and estrogen [63], promote EPC migration and proliferation and suppress EPC senescence and apoptosis. Among the aforementioned mechanisms, the activation of the SDF-1/CXCR4/PI3K/Akt axis most significantly influences EPC migration.

#### Adhesion

There is limited data about the mechanisms of EPC adhesion at neovascularization sites, although recent findings indicate that platelets play a role in EPC adhesion to injury sites and that this process mainly relies on the interaction between P-selectin glycoprotein ligand-1 (PSGL-1) on EPCs and P-selectin on platelets [64,65]. Platelets are activated upon vascular injury-induced exposure of the subendothelial layer, and this process involves the up-regulation of P-selectin expression and the increased SDF-1 release into neovascularization sites. The released SDF-1 and its receptor CXCR4 play a key role in EPC recruitment and adhesion to ischemic areas [66]. Furthermore, PSGL-1 and its main ligands (P, E, and I-selectin) are cell adhesion molecules that regulate initial interactions between leukocytes and blood vessel walls, as well as between activated platelets and EPCs [64,67,68]. Additionally, in EPCs, CXCR7 also interacts with lymphocyte function-associated antigen-1 (LFA-1) and very late antigen-4 (VLA-4) to regulate the capacity of EPCs to adhere to ECs in ischemic tissue [69]. Integrins, a superfamily of cell adhesion molecules, regulate EPC functions by binding to the extracellular matrix, cell-surface proteins, and soluble ligands [70]. Various integrin subunits, including α1, α2, α3, α4, α5, α6, α9, αv, β1, β2, β3, β5, and β7 regulate different steps of EPC biological functions [70], and β2 integrin plays a key role in EPC adhesion to neovascularization sites. β2 integrin mediates the adhesion of PB-derived EPCs to pre-activated EC monolayers, as well as ICAM-1 and fibrinogen. Additionally, the same study also revealed that β2 integrin plays an essential role in the homing of BM-derived EPCs to ischemic tissues, as well as in EPC neovascularization capacity *in vivo* [71]. Cyclic AMP activation in EPCs can increase the adhesion of β2-integrin to ICAM-1 and promote the homing of intravenous EPCs, thereby increasing new blood vessel formation [71–73]. The PI3K/Akt signaling pathway is also thought to influence EPC homing to the neovascularization sites and to improve blood perfusion via α4-integrin [74,75]. EPCs have been shown to bind to ECs through the TNF-α-regulated VCAM-1/VLA4 interaction in injured tissues [76]. ECs may also act as antigen-presenting cells between the ECs in microvessels and T cells. When compared with ECs on the walls of blood vessels, MHC-II expression is significantly elevated in detached ECs, which enables EC recognition by T-cell receptors and T-cell activation and proliferation [77]. T cells undergo a sequence of interactions with EPCs through the expression of adhesion molecules, including ICAM-1 or VCAM-1, which promotes EPC adhesion to neovascularization sites, thereby accelerating tissue repair.

#### Differentiation

EPCs have the potential to differentiate into various cell types, such as mature ECs, fibroblasts, hepatocytes, and neurocytes. When compared with CD34+/CD133+ cells, CD34-/CD133+ progenitor cells exhibit a significantly higher capacity to differentiate into non-EC cell types, such as fibroblasts, hepatocytes, cardiomyocytes, and neurocytes [78]. EPCs participate in neovascularization through the interdependent processes of differentiation into mature ECs to directly form new blood vessels and the paracrine promotion of interactions between pre-existing ECs and other cell types. Platelets are critical for EPC recruitment to ischemic lesions and for the induction of EPC differentiation into ECs as revealed by the expression of EC differentiation markers, such as vWF and CD31 [79]. Furthermore, EPC co-incubation with platelets induces their migration and colonization of platelet thrombi [80]. VEGF can up-regulate Flk-1 expression and induce EPC differentiation into mature ECs, and Flk-1 can also regulate the proliferation, migration, chemotaxis, and survival of mature ECs [81,82]. A recent study found that co-culturing EPCs with VEGF-secreting bone marrow mesenchymal stem cells (BMSCs) enhanced the expression of the endothelial markers, indicating that BMSCs regulate EPC differentiation via VEGF [83]. More specifically, VEGF promotes EPC differentiation through various signaling pathways, including PI3K/Akt, ERK, eNOS, Wnt, mammalian target of rapamycin, and hypoxia-inducible factor-1α (HIF)-1α [81,84–87].

### EPCs in pregnancy

#### EPCs in maternal PB

Initially, Junichi Sugawara et al. reported that increased cEPC levels correlated positively with estrogen levels [88], that estrogen promotes EPC mobilization from the BM *in vivo*, and that it inhibits EPC senescence by up-regulating VEGF production *in vitro* [89]. Subsequent studies used flow cytometry to show that in normal pregnancies, the number of cEPCs increased within weeks of gestation and that the number of EPCs was higher in the third trimester when compared with the postpartum period [90,91]. However, findings by Matsubara et al. indicated EPC elevation in early pregnancy and a decrease in mid-late pregnancy, which was thought to coincide with a peak in angiogenesis [92]. In normal pregnancies, EPCs from twin pregnancies are higher than in singleton pregnancies [92]. However, Attar et al. reported the opposite observation, that CFU-ECs fall in early and mid-pregnancy and rise in late pregnancy [93], whereas Parsanezhad et al. found no significant changes in the levels of CFU-ECs and ECFCs in pregnant vs. non-pregnant cases [94]. The reasons for these discrepancies are unclear and need further investigation.

#### EPCs in UCB

There is ongoing debate about the quantity of EPCs in UCB. The number of UCB-derived ECFCs is reported to be higher in preterm versus in term infants, suggesting that the number of EPCs might affect gestational weeks [95]. However, other studies found no significant differences in different gestational weeks [96–99]. The different view was that the number of EPCs increases with advancing gestational age. They found that the number of UCB-derived ECFCs was constant between gestational weeks 24–31, 2-fold at 32–36 weeks, and 3-fold at 37–40 weeks [100]. Decreases in CB-derived ECFC were observed in patients with various pregnancy-associated complications, such as GDM, PE, and FGR [101,102].

#### Origins of EPCs in circulation of pregnancy

Studies have shown that the obtained CFU-ECs and ECFCs in maternal PB originated from mothers rather than their fetuses due to the loss of fetal Sexdetermining Region Y (SRY) gene in CFU-ECs and ECFCs [103,104]. Significantly, in maternal PB, some CD34+ cells which derived from ECs rather than hematopoietic lineages were from the fetuses [105]. To date, these fetal progenitor cells cannot be proved to be EPCs, but they displayed similar EPCs’ behaviors, entering the maternal circulation and participating in maternal physiological and pathological activities, such as uterine and placental vascularization and enhancement of placental perfusion, during pregnancy [106,107].

#### EPCs in the placenta

Placenta-derived eCFCs have been detected in placental microvasculature and macrovasculature and are thought to have different functions and origins [108]. Placental macrovascular eCFCs are fetus-derived, whereas the placental microvasculature is derived from the mother. Moreover, fetus-derived placental eCFCs express the same cell surface markers as UCB-derived eCFCs and they can also enter into the maternal circulation [109]. Additionally, the gene expression and angiogenic capacity of placenta- and UCB-derived eCFCs do not differ significantly *in vivo* and *in vitro* [110]. Therefore, it is still unclear whether placental eCFCs are the source of fetal- or maternal-derived EPCs [93].

## The role of EPC in pregnancy-associated disorders

### EPCs and PE

#### The number of EPCs in PE

PE is a pregnancy hypertensive disorder that affects 2–8% of pregnancies worldwide. PE is categorized as early (before 34 weeks of gestation) or late (at or after 34 weeks of gestation) onset [111]. When compared with late-onset PE, early-onset PE is associated with a higher rate of severe complications, including severe PE, fetal growth restriction, and neonatal death [112]. Current evidence indicates that early- and late-onset PE have different pathophysiologic mechanisms. Early-onset PE is associated with insufficient remodeling of uterine spiral arteries, placental ischemia, and placental dysfunction, whereas late-onset PE is associated with maternal systemic inflammation [113]. However, both subtypes are associated with maternal endothelial dysfunction. Studies suggest that using angiogenesis-related biomarkers, such as the sFlt/PLGF ratio is more effective at predicting early-onset PE than late-onset PE [112,114]. The pathophysiology of placental and maternal endothelial dysfunction is multifactorial and includes oxidative stress, vasoconstrictors, anti-angiogenic factors, and inflammatory cytokines [56,115]. These factors may also affect the distribution of the EPC pool, thereby altering the number of circulating EPCs in pregnancy-related diseases. Table 2 summarizes literature describing changes in EPC numbers in pregnancy-related disorders.

**Table 2:** Diseases characterized by alteration in EPCs number

Study	Diseases	EPCs origin	Effect on EPCs number	Pathway
Dirong Dong [116]	PE	PB	Decrease	MicroRNA-646/VEGF-A/HIF-1α
Ji-Hee Kim [101]	PE	PB; UCB	Decrease	TNF-α/NF-κB/miR-31/155/ eNOS/NO
Nicole Brown [117]	PE	PB	Increase	VE-Cadherin
Yejin Park [118]	PE	UCB	Decrease	H3K4 and H3K9 trimethylation
Yangui Wang [61]	GH, PE	PB	Decrease	Unknown
Antonio Simone [119]	PE	PB	Decrease	Unknown
Ying Hu [120]	PE	UCB	Decrease	Axl
Xiaoxia Liu [121]	PE	UCB	Decrease	Notch1
Mohammad-Ebrahim [94]	PE	PB	Increase	Unknown
Ting Yan [122]	PE	UCB	Decrease	Mir-126/PIK3/Akt
Ranjan Monga [99]	PE, IUGR	UCB	Decrease	Unknown
Patrizia Luppi [90]	PE	PB	Decrease	Unknown
Carol Lin [104]	PE	PB	Decrease	Unknown
Ja-Young Kwon [123]	PE	UCB	Decrease	VEGF
Junichi Sugawara [124]	PE	PB	Decrease	C-reactive protein
Yuehuan Wu [102]	GDM	UCB	Decrease	PI3K/AKT/eNOS
Panagiota Markopoulou [125]	GDM	PB	Decrease	Unknown
U Deniz Dincer [126]	GDM	UCB	Decrease	VEGFA, HIF1α
Giuseppe Penno [127]	GDM	PB	Decrease	Unknown
David A Ingram [128]	GDM	UCB	Decrease	Unknown
Michele Buemi [129]	GDM	PB	Decrease	Unknown
Mariane Bertagnolli [130]	Preterm birth	PB	Decrease	Unknown
Marina Podestà [97]	Preterm birth	UCB	No change	Unknown
Paula Frizera Vassallo [131]	Preterm birth	UCB	Unknown	SIRT1
Alessandro Borghesi [132]	Preterm birth	UCB	Decrease	Unknown
Christopher D Baker [133]	Preterm birth	UCB	Increase	Unknown
V Oliveira [134]	IUGR	PB; BM	No change	eNOS/NO
F Calcaterra [135]	IUGR	PB	Decrease	PGF and SDF-1
Peter I Sipos [136]	IUGR	UCB	Decrease	SDF-1 and MMP2
Thomas F J King [137]	IUGR	PB	Decrease	Unknown
Han Sung Hwang [138]	IUGR	UCB	Decrease	Telomerase activity
Simin Asadian	PROM	PB	Increase	Oestrogen
Tomohisa [139] Sakashita [140]	Placental abruption	PB	Decrease	sFlt-1, PlGF
Kazuyoshi Kanki [141]	Miscarriages	BM	No change	sFlt-1, SDF-1, eNOS

BM, bone marrow; EPC, endothelial progenitor cell; GDM, gestational diabetes mellitus; GH, gestational hypertension; IUGR, intrauterine growth restriction; PB, peripheral blood; PE, preeclampsia; PROM, premature rupture of membranes; UCB, umbilical cord blood.

The number of EPCs in PB is reported to be significantly reduced in patients with PE when compared with normal pregnant women and to positively correlate with the levels of angiogenic T cells. However, other studies indicate that the number of CD34+/VE-cadherin+ EPCs in PB is significantly higher in patients with PE than in controls and that this correlated positively with systolic blood pressure [117]. Moreover, a significant reduction in the number of EPCs has been observed in CB samples of preeclamptic patients [142–144]. Young Kwon et al. found that CB-derived EPCs and cord plasma-free VEGF-A are significantly reduced in patients with severe PE [123]. Therefore, it is likely that the fluctuation in the number of PB-derived EPCs depends on the PE stage, with EPC numbers decreasing at PE onset and then increasing at the compensatory stage. Moreover, PE is often accompanied by other pregnancy complications, which may cause the number of EPCs to fluctuate. Pathways and changes in EPC numbers during PE are shown in Table 2.

#### Regulation of EPC function in PE

##### Neurogenic notch homolog protein (Notch) and VEGF pathways

Notch signaling is a key regulator of various cellular functions. There are four Notch receptors (Notch 1–4) and the pathways influence vascular remodeling and stabilization. In EPCs, the Notch pathway might inhibit cell proliferation, differentiation, migration, and tube formation. In PE, Notch4 signaling, but not Notch1, inhibits EPC function via the DLL4/Notch/ephrin-B2 pathway [145]. However, it is reported that in EPCs, histone-lysine N-methyltransferase (G9a), which controls the transcriptional switch, is activated by the Notch/RBP-J pathway to promote vascular maturation in extraembryonic tissues and is required for embryonic survival [146]. In response to vascular injury, EPCs rapidly migrate to injured sites and form new vessels by directly differentiating into ECs and indirectly secreting vascular regulatory factors, including VEGF, SDF-1, and NO. Therefore, an understanding of EPC-induced changes to vascular regulatory factors during PE may improve our understanding of PE pathogenesis and overcome the current challenges to PE treatment using EPC cell therapy. UCB-derived EPC levels have been reported to be markedly reduced in PE and this is accompanied by a significant decrease in cord plasma VEGF-A levels [123]. Along with neuropilin-1 and VEGFR-2 (KDR), VEGF promotes EPC survival [147]. sFlt-1 is mainly produced in the placenta and acts as a decoy receptor by binding to VEGF-A and PlGF, thereby reducing their availability to target cells. The sFlt-1/PlGF ratio has been reported as a useful tool for the prediction of PE and adverse pregnancy outcomes [148]. In PE, the number of UCB-derived EPCs correlates inversely with sFlt-1 levels and sFlt-1 significantly inhibits the migration and proliferation of EPCs by suppressing the expression of eNOS and SDF-1 [141,144,149]. Kim et al. reported that elevated serum TNF-α in preeclamptic patients inhibits EPC function by down-regulating eNOS expression [101]. Ang II negatively modulates vascular regeneration by binding to Ang II receptor subtype 1 (AT1R) and reducing the number and function of EPCs [150]. Keiichi Matsubara et al. found that Ang II and TNF-α suppress EPC mobilization into the systemic circulation, which may impair EC regeneration and neovascularization in PE [151].

##### miRNA and epigenetics

MicroRNAs (miRNAs) are small noncoding RNAs that regulate gene expression by recognizing cognate sequences and interfering with transcriptional, translational, or epigenetic regulation. The miRNA-mediated regulation of EPC function during PE has been extensively studied. For example, UCB-derived EPCs of preeclamptic patients showed that at cell culture passages 3 and 7 (P3 and P5) 17 and 47 miRNAs were significantly differentially expressed, respectively, when compared with control cells. In PB-derived EPCs from the PE group, 39 and 17 miRNAs were significantly differentially expressed in P3 and P5, respectively, when compared with the controls [152]. The target genes of these miRNAs were mainly associated with the EPC cycle, adhesion, and selective shearing processes. Moreover, Hsa-miR-1270 levels were elevated in UCB-derived ECFCs from patients with PE, which was accompanied by the up-regulation of its target genes ANGPTL7 and TFRC, resulting in reduced ECFC tube formation and chemotactic motility [152]. However, Bianca Schröder-Heurich et al. observed decreased miR-1270 levels in UCB-derived EPCs from PE patients and reported that its dysregulation contributed to vascular dysfunction through the ataxia telangiectasia mutated (ATM)/tyrosine kinase Src/VE-cadherin pathway [153]. In preeclamptic patients, miR-126 levels were significantly reduced in UCB-derived EPCs, which exerted pro-angiogenic functions through the PIK3R2/PI3K/Akt pathway [122]. The effect of miR-646/VEGF-A/HIF-1α signaling axis and the miR-483/PI3K/Akt pathway is significant for angiogenic properties of EPCs in placental vasculogenesis in PE [116,154].

DNA methylation is reported to influence EPC function and vascular repair in PE. Analysis of cultured UCB-derived ECFCs from PE patients detected 1266 and 2362 CpG methylation sites at P3 and P5, respectively. These sites were different from those detected in the control group and that they were associated with DNA methylations that are affected by metabolism, the cell cycle, and transcription [155]. Moreover, PE was associated with lower trimethylation levels on histone H3K4 and H3K9 in UCB-derived EPCs, which significantly delayed differentiation and suppressed EPC colonies and functions, including migration, adhesion, and tube formation [118].

##### Cellular senescence

**Figure 3:**
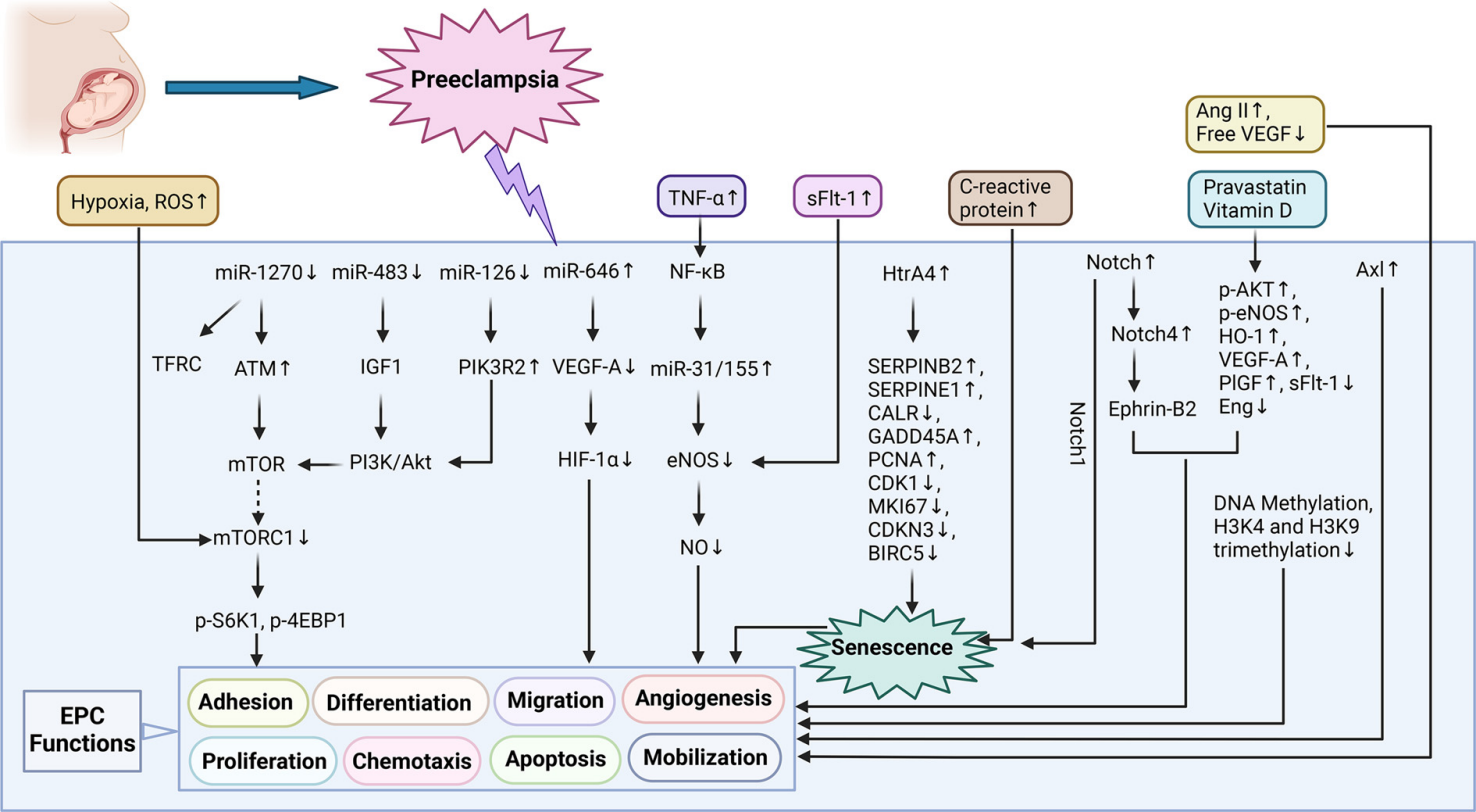
Mechanisms about the regulation of EPCs function in preeclampsia

Cellular senescence is induced by various types of stress that cause irreversible cell cycle arrest and distinct cellular dysfunctions. Stem cell senescence is characterized by reduced cell regeneration and self-renewal, which affects vascular remodeling. Hypertension can accelerate EPC senescence via mitochondrial dysfunction, antioxidant reduction, and the AngII/HO-1/NO, CXCR4/JAK2/SIRT5, and aldosterone/SIRT1/p53/p21 pathways, which further impair EPC function [156–162]. Therefore, determining the senescence mechanism of EPCs may elucidate PE pathogenesis. For example, in PE, increased C-reactive protein (CRP) serum levels are reported to significantly down-regulate EPC colony formation and function [124]. In PE, sFlt-1 levels in UCB have also been correlated with UCB-derived EPC senescence, suggesting that EPC dysfunction is probably associated with increased sFlt-1 levels [144]. The factors affecting EPC function in PE are shown in Table 3 and Figure 3.

**Table 3:** Factors affecting EPC functions in PE

Factors	Mode of action	Functions	References
miRNA	miR-1270↓/ATM↑/mTOR/Src/VE-cadherin; Hypoxia, ROS↑/mTORC1↓/p-S6K1, p-4EBP1	Chemotaxis, angiogenesis, and adhesion	[153]
	miR-1270↓/ TFRC	Proliferation, chemotaxis, and angiogenesis	[152]
	miR-483↓/ IGF1/PI3K/Akt/mTOR	Adhesion and angiogenesis	[154]
	miR-646↑/VEGF-A↓/HIF-1α↓	Proliferation, differentiation, and migration	[116]
	miR-126↓/PIK3R2↑/PI3K↓/Akt↓	Proliferation, differentiation, migration, and angiogenesis	[122]
	TNF-α↑/NF-κB/miR-31/155↑/eNOS/NO↓	Mobilization, differentiation, and angiogenesis	[101]
Methylation	DNA Methylation↑	Angiogenesis	[155]
	Histone methylation: H3K4 and H3K9 trimethylation↓	Differentiation, migration, adhesion, and angiogenesis	[118]
Drugs	Pravastatin/p-AKT↑, p-eNOS↑, HO-1↑, VEGF-A↑, PlGF↑, sFlt-1↓, Eng↓	Proliferation, migration, and angiogenesis	[149]
	Vitamin D	Migration and angiogenesis	[60]
Senescence	HtrA4↑/SERPINB2↑, SERPINE1↑, CALR↓, GADD45A↑, PCNA↑	Apoptosis and senescence	[163]
	HtrA4↑/CDK1↓, MKI67↓, CDKN3↓, BIRC5↓	Proliferation and differentiation	[164]
	C-reactive protein↑	Senescence	[124]
Notch pathways	Notch1↑	Proliferation, differentiation, migration, and adhesion	[121]
	Dll4/Notch↑/ Ephrin-B2	Proliferation, differentiation, migration, and angiogenesis	[165]
	DLL4/Notch4↑/EFNB2↑	Proliferation, differentiation, migration, adhesion, and angiogenesis	[145]
Other factors	Axl↑	Proliferation, differentiation, migration, and adhesion	[120]
	sFlt-1↑	Proliferation, migration, and angiogenesis	[144]
	sFlt-1↑/eNOS↓	Proliferation, migration, and angiogenesis	[141]
	Ang II and TNF-α↑	Proliferation and angiogenesis	[151]
	Free VEGF↓	Angiogenesis	[123]

EPC, endothelial progenitor cell; PE, preeclampsia.

### EPCs and GDM

GDM is associated with vascular endothelial dysfunction in fetoplacental macrovessels and microvessels [166]. Patients with GDM exhibit lower numbers of UCB-derived EPCs at gestation weeks 24–32 and 1–2 days after delivery [91]. Buemi et al. found that patients with GDM have more CD133+/VEGFR2+ cells when compared with women with normal pregnancies [129]. Juan et al. found that EPC subtypes vary differently in women with GDM, who exhibited lower cEPC levels without changes in CFU-ECs [91]. Although studies of the effects of ECFCs in GDM have yielded contradictory findings, *in vitro* studies show that generally, proliferation, migration, and tube formation are impaired [34,167]. In addition, it is reported that exposing ECFCs to a hyperglycemic environment during GDM induces unique phenotypic alterations and that ECFCs enhance resistance to hyperglycemia-induced senescence through decreased p38MAPK activation [168]. Epigenetic processes, such as DNA methylation, are the most widely studied GDM-altered processes. The expression of placenta-specific 8 (PLAC8) is elevated in UCB-derived ECFCs from patients with GDM because of decreased DNA methylation at specific PLAC8 CpG sites. High PLAC8 expression in ECFCs is reported to promote proliferation and delay senescence, which may be an adaptive, protective fetal response [169].

### EPCs and preterm birth

Preterm birth is associated with vasculopathy, which can increase the risk of prematurity-related complications, such as bronchopulmonary dysplasia and retinopathy of prematurity. EPCs are involved in the vascular pathophysiology of prematurity-related disorders. Studies found that in preterm infants, the numbers of EPCs correlate negatively with APGAR scores and that EPC counts positively correlate with the risk of respiratory distress syndrome, retinopathy, bronchopulmonary dysplasia, patent ductus arteriosus, and infections [96]. However, Christopher et al. found that when compared with term ECFCs, preterm ECFCs are elevated, and that they proliferate more rapidly, with greater susceptibility to hyperoxia, which can be alleviated by antioxidants, such as superoxide dismutase and catalase [133]. Oxidative stress because of high oxygen levels (40%) inhibits ECFC proliferation, induces senescence, and impairs angiogenesis by inhibiting Notch signaling [98] and disrupting the VEGF/eNOS/NO pathway [170].

### EPCs and IUGR

Abnormal placental vascular function can trigger IUGR, which can also impair the modulatory ability of fetal vascular ECs, leading to an increased risk of long-term cardiovascular diseases. When compared with PB-derived EPCs from normal pregnant women, PB-derived EPCs from patients with IUGR exhibit impaired function, which may be associated with significantly lower circulating levels of PIGF and SDF-1 [135]. When compared with healthy fetuses, lower levels of ECFCs, with lower capacities *in vitro* and *in vivo*, have been isolated from fetuses with IUGR, probably because of unbalanced antiangiogenic factors (e.g., thrombospondin-1) and proangiogenic factors (e.g., MMP-2 and SDF-1) [136,171]. Furthermore, it is reported that fetuses with IUGR exhibit early changes in vascular structure and functions and are more likely to suffer from long-term cardiovascular-related diseases [172]. Fetal EPCs from FGR-affected pregnancies exhibit prolonged differentiation times, as well as markedly few EPC colonies and significantly higher senescence because of decreased telomerase activity [138]. Therefore, the number and function of maternal and fetal EPCs may be a promising biomarker for pregnancy monitoring.

### EPCs and other pregnancy diseases

When compared with women with term pregnancies, those with premature rupture of membranes exhibit a higher number of cEPCs accompanied by higher levels of serum estrogen, VEGF, and SDF-1. *In vitro* analysis revealed enhanced EPC function, which may be associated with a higher expression of estrogen receptor alpha (ER-α). 17β-Estradiol promotes EPC proliferation, migration, and tube formation [139]. In addition, a mouse model of recurrent abortion revealed higher levels of sFlt-1in PB and that EPC functions were reduced via the sFlt-1/VEGF–PI3K/Akt–eNOS pathway. However, this dysfunction was ameliorated by the homing of exogenous, transfused EPCs in the placenta [141]. Moreover, when compared with patients with PE but without placental abruption, the number of EPCs is reported to be markedly reduced with increased sFlt-1 and decreased PIGF levels in women with PE and placental abruption [140]. This suggests that the number of EPCs can predict placental abruption in patients with PE. However, studies are needed to further elucidate the role of EPCs in pregnancy-associated diseases. Tables 2 and 4 show factors that affect EPC functions and numbers during pregnancy diseases.

**Table 4:** Factors affecting EPC functions in other pregnancy diseases

Diseases	Factors	Functions	References
GDM	Chronic hypoxia/eNOS↓, P-eNOS↑, HIF 1α↑, IGF-1↑	Senescence and angiogenesis	[126]
	TAGLN↑	Migration and angiogenesis	[173,174]
	Vitamin D3/Vitamin D receptor	Migration and angiogenesis	[167]
	Bradykinin/PI3K/Akt↑/eNOS↑ signalling pathway, hTERT translocation↓, ROS↓	Senescence	[102]
	Moderate hypoxia/eNOS↓, RAMP↓	Senescence and angiogenesis	[175]
	Hyperglycemia/p38MAPK↑	Proliferation, senescence, and angiogenesis	[168]
	Hyperglycemia/ PLAC8↑	Proliferation and senescence	[169]
Premature birth	Superoxide dismutase, Catalase	Proliferation	[133]
	Hyperoxia/ VEGFR2↓/eNOS/NO↓	Proliferation	[170]
	THBS1↑/p-AKTC	Migration, angiogenesis, and proliferation	[171]
	EPO↑, IGF-1↓	Mobilization	[176,177]
	RSV, SIRT1↓	Senescence, proliferation, and angiogenesis	[131]
	SIRT1↓/MKK6/p38MAPK/Hsp27 pathway	Senescence	[178]
IUGR	NO↓, p-eNOS↑	Senescence	[134]
	G9a↓/Notch	Proliferation	[179]
	SDF-1↓, MMP-2↓	Angiogenesis	[136]
PROM	oestrogen↑	Migration, angiogenesis, and proliferation	[139]
Recurrent miscarriage	sFlt1↑/VEGF/PI3K/Akt/eNOS↓	Angiogenesis	[141]

EPC, endothelial progenitor cell; GDM, gestational diabetes mellitus; IUGR, intrauterine growth restriction; PROM, premature rupture of membranes.

## EPC-based therapies for pregnancy-related diseases

Regenerative medicine, which includes cell therapy and tissue engineering, is a multidisciplinary field that seeks to replace, repair, or restore injured tissues. Because of their unique properties, postpartum tissues like the placenta, umbilical cord, and UCB, are well-suited for regenerative medicine [180]. For example, stem cells from postpartum tissues have received significant attention because they are easily accessible and relatively safe for the fetus [181,182]. Among the candidate stem cells, EPCs have received a lot of attention and are expected to be candidates for cell therapy.

### Diagnostic tools

A case–control analysis found that during the first trimester, the number of EPCs was significantly lower in the PE group when compared with the uncomplicated pregnancy group and that the evaluation of EPCs in PB during the first trimester might be an effective method for the early detection of women who may be at risk of developing PE [119]. In addition, the rate of cEPCs senescence was significantly higher in patients with PE when compared with the control group. EPC colony counts are reported to be markedly lower in CRP-positive preeclamptic patients when compared with CRP-negative preeclamptic patients [124]. However, these experiments have not investigated the sensitivity and specificity of EPCs in predicting pregnancy-related diseases. Further basic and clinical trials are needed to validate the sensitivity and specificity of EPCs as predictors of pregnancy-associated disorders.

### Therapeutic strategies

Directly injecting EPCs into the circulation of mother or the placenta is effective and safe in animal models. Specifically, EPC transplantation can promote placental angiogenesis, lower physiological parameters like proteinuria and blood pressure to normal levels, and relieve fetal brain hypoxia by suppressing nestin expression in a rat model of PE [183]. Moreover, Reshef Tal et al. found that transfused EPCs homed to the placenta and normalized its vascular structure by up-regulating IGF-2 and VEGF and was associated with a reduced rate of miscarriage [141]. To further improve the efficacy of EPCs, various studies have investigated the role of EPCs in placental angiogenesis after treatment using drugs or gene editing. Estrogen is reported to increase cEPC numbers by suppressing apoptosis and to restore their functions by affecting NO and ROS production. The levels of serum estradiol and cEPC numbers have been shown to exhibit a significant positive correlation, suggesting that estrogens may exert vasoprotective effects during human pregnancy [88]. 17β-Estradiol is reported to restore the biological features of EPCs from pregnant women with premature rupture of membranes [139]. Additionally, a maternal high-fat diet is thought to contribute to adverse cardiovascular diseases in mice offspring, which may be caused by reduced cEPC levels [184]. Mounting evidence shows that statins promote EPC function while reducing their senescence and apoptosis. For example, lovastatin can reverse impaired EPC function via the Akt/eNOS signaling pathway in a high-fat environment [185]. Pravastatin is reported to enhance cEPC numbers and reduce CRP levels in mice offspring, which might lower the future risk of cardiovascular disease [184]. During gestational hypertension and PE, antihypertensive drugs, such as metoprolol, methyldopa, and nifedipine decrease systolic and diastolic blood pressure and enhance the number and function of EPCs, highlighting a pharmacological mechanism for the treatment of hypertensive disorders [61]. Vitamin D deficiency has been shown to play a key role in the pathophysiology of PE by regulating the secretion of VEGF by EPCs, and vitamin D supplementation reverses the adverse effects on placental angiogenesis in PE [186,187]. Drug-loaded liposomal nanoparticles, which have controlled drug delivery can be used to modify EPCs. Specifically, combining EPCs with nanoparticles is proposed as a way of improving vasculogenesis with the potential to cure GDM [173]. Insulin has been previously shown to promote EPC mobilization from the BM to the PB in diabetic animals and adults. The association between maternal insulin therapy and high fetal CD34+/KDR+ cell counts remained significant in GDM, suggesting that insulin increased the number and function of EPCs by elevating VEGF production in the fetal circulation [188]. Table 5 summarizes EPC-based therapies for pregnancy-related diseases.

**Table 5:** Studies investigating EPCs-based therapies for pregnancy-related diseases

Treatments	Diseases	EPCs Origin	Effects	Pathways	Research objects	References
Direct EPCs transplantation	PE	PB	Placental angiogenesis↑; Proteinuria↓; Blood pressure↓; Intrauterine hypoxia↓	Nestin	Rat	[183]
Direct EPCs transplantation	Miscarriage	BM	Miscarriage↓; Placental angiogenesis ↑	VEGF/eNOS	Mouse	[141]
Estradiol	PROM	PB	Anti-apoptosis↑; Proliferation↑; Migration↑; Angiogenic secretory activity↑	NO and ROS	Human	[139]
Pravastatin	Hyperlipidemia	BM	EPC number↑; Colony numbers↑	C-reactive protein	Mouse	[184]
Metoprolol, methyldopa, and nifedipine	GH, PE	PB	EPC number↑; Colony formation capacity↑; Proliferation↑; Migration↑	Unknown	Human	[61]
Vitamin D3	PE	UCB	Tube formation↑; Differentiation↑; Invasion↑	VEGF VE-cadherin	Human	[186,187]
Insulin	GDM	UCB	EPC number↑;	Unknown	Human	[188]
Relaxin	PE, GDM	BM	Migration↑; Tube formation↑	PI3K/Akt/NO	Mouse	[189]
Transplantation EPC treated with platelet microparticles	PE	PB	Blood pressure↓; Proteinuria↓; Placental angiogenesis↑	eNOS/NO	Rat	[190]

BM, bone marrow; EPC, endothelial progenitor cell; GDM, gestational diabetes mellitus; GH, gestational hypertension; PB, peripheral blood; PE, preeclampsia; PROM, premature rupture of membranes; UCB, umbilical cord blood.

However, further investigation was needed for the unknown area. First, reduced cEPC level was observed in many pregnancy-related complications and could predict their risks as a biomarker. Therefore, cEPC evaluation is encouraged to aid in the diagnosis and early intervention of complications. Second, the definition of EPCs was ambiguous, and precise definition is needed to facilitate their widespread application as a prognostic factor in pregnancy-related diseases. Third, although many drugs could improve EPCs’ functions and numbers during pregnancy, the specific mechanisms, appropriate dosage and safety remain to be explored. Finally, it should be noted that some mouse and rat experiments might not translate to humans because of differences in immune function, as well as differences in the uteroplacental structure and placental type [191]. Nonetheless, because some initial observations cannot be studied in pregnant women, animal models are a critical tool in this area of research. Although numerous studies have been conducted in animal models, few treatments have entered clinical trials, highlighting the long process of translating animal findings into human therapies for pregnancy-associated diseases.

## Conclusion

Disorders that affect neovascularization during pregnancy can cause pregnancy-associated diseases. EPCs, the precursors of ECs, are impaired in many diseases and numerous studies have investigated their potential as therapeutic targets for the treatment of pregnancy disorders. This review describes the discovery, origin, isolation, and definition of EPCs based on cell surface markers, shape, and functions. The functions of EPC, such as mobilization, migration, adhesion, and differentiation, are regulated by many pathways, including VEGF, SDF-1/CXCR4, and PI3K/Akt/eNOS/NO signaling. We have also summarized the changes in the numbers and functions of EPCs, as well as pre-clinical studies of potential diagnostic and therapeutic strategies that can target EPCs against pregnancy-associated disorders, such as PE, GDM, and IUGR. However, knowledge of the role of EPCs in neovascularization remains limited. EPCs have important implications for regenerative medicine and further research is required for effective translation into clinical applications.

## Data Availability

Not applicable (review article)

## Abbreviations

Ang II: Angiotensin II
AT1R: Ang II receptor subtype 1
ATM: ataxia telangiectasia mutated
BM: bone marrow
BMSC: bone marrow mesenchymal stem cell
CAC: circulation angiogenetic cell
CEC: circulating endothelial cell
cEPC: circulating EPC
CFU-EC: colony forming unit-endothelial cell
CRP: C-reactive protein
CXCR4: CXC-chemokine receptor 4
DGC: density gradient centrifugation
EC: endothelial cell
ECFC: endothelial colony-forming cell
ECM: extracellular matrix
EC: endothelial cell
eEPC: early EPC
eNOS: endothelial nitric oxide synthase
EPC: endothelial progenitor cell
ER-α: estrogen receptor alpha
ET: endothelin
FACS: fluorescence activated cell sorting
FGF: fibroblast growth factor
Flk-1: foetal liver kinase 1
GDM: gestational diabetes mellitus
GH: gestational hypertension
HIF-1α: hypoxia-inducible factor-1α
HSC: hematopoietic stem cell
ICAM-1: intercellular cell adhesion molecule-1
IL: interleukin
IMI: immunomagnetic isolation
IUGR: intrauterine growth restriction
KDR: kinase insert domain receptor
lEPC: late EPC
LFA-1: lymphocyte function-associated antigen-1
miRNA: microRNA
mkitL: membrane bound kit Ligand
MMP-9: matrix metalloproteinase-9
MMP: matrix metalloproteinase
MNC: mononuclear cells
mTOR: mammalian target of rapamycin
NO: nitric oxide
Notch: neurogenic notch homolog protein
OEC: outgrowth endothelial cell
PB: peripheral blood
PE: preeclampsia
PLAC8: placenta-specific 8
PlGF: placenta growth factor
PROM: premature rupture of membranes
PSGL-1: P-selectin glycoprotein ligand-1
SDF-1: stromal cell-derived factor-1
sFlt-1: soluble fms-like tyrosine kinase 1
sKitL: soluble kit ligand
SRY: sex-determining region Y
TNF-α: tumor necrosis factor-α
UCB: umbilical cord blood
VCAM-1: vascular cell adhesion molecule-1
VEGFR2: vascular growth factor receptor 2
VLA-4: very late antigen-4
